# The Complete Genome Sequence of *Amorphophallus titanum,* the Corpse Flower

**DOI:** 10.56179/001c.37841

**Published:** 2022-08-12

**Authors:** Linda Frisse, Marc A Martinez, Stacy Pirro

**Affiliations:** 1 National Center of Biotechnology Information, National Institutes of Health; 2 Carnivero; 3 Iridian Genomes

**Keywords:** genome, viridiplantae, corpse flower

## Abstract

The Corpse Flower, or Titan Arum (*Amorphophallus titanum*) is a flowering plant in the family Araceae. endemic to a limited range in the rainforests of Sumatra, Indonesia. It is notable for two reasons: it produces the world’s largest known unbranched flower, and it produces a strong odor of rotting meat to attract pollinators. We present the whole genome sequence of this species. A total of 335,712,220 paired-end Illumina reads consisting of 100.7G bases were obtained by Illumina sequencing the leaf tissue of a single individual. The reads were assembled by a *de novo* method followed by contig extension using related species as references. The raw and assembled data is publicly available via Genbank: Sequence Read Archive (SRR11565159) and genome assembly (GCA_024336825).

## Introduction

*Amorphophallus titanum* is notable both for the large size and strong smell of its flower. The species does not have an annual flowering cycle, but instead produces a bloom every several years, although some individuals produce a bloom on a shorter cycle. The flower can reach over 10 ft (3m) in height and consists of a fragrant spadix of flowers wrapped by a spathe, which looks like a large petal. The spathe is a deep green on the outside and dark burgundy red on the inside, with a deeply furrowed texture.

When in bloom, the flower has an extremely strong odor of rotting meat. The smell attracts insects that normally feed on dead animals and these act as pollinators. The species has male and female forms, and is also capable of selfpollination. Self-pollination has occurred spontaneously in botanical garden settings when there has only been a single specimen present, and also manually by horticultural staff.

The species is endemic to a small region in Sumatra, Indonesia. Because of shrinking habitat, the species is considered endangered in the wild.

## Methods

A single live cultivated plant was used for this study. The specimen was obtained from Carnivero (Austin, Texas, USA).

DNA extraction was performed using the Qiagen DNAeasy genomic extraction kit using the standard process. A paired-end sequencing library was constructed using the Illumina TruSeq kit, according to the manufacturer’s instructions. The library was sequenced on an Illumina Hi-Seq platform in paired-end, 2 × 150bp format. The resulting fastq files were trimmed of adapter/primer sequence and low-quality regions with Trimmomatic v0.33 (Bolger, Lohse, and Usadel 2014). The trimmed sequence was assembled by SPAdes v2.5 (Bankevich et al. 2012) followed by a finishing step using Zanfona v1.0 (Kieras 2021) to make additional contig joins based on conserved regions in related species.

## Results

The genome assembly yielded a total sequence length of 942,822,506 bp over 9,603 scaffolds with an N50 of 15.37 MB.

## Data Availability

Raw and assembled data is publicly available via GenBank: raw genome data https://trace.ncbi.nlm.nih.gov/Traces/sra/?run=SRR11565159 assembled genome https://www.ncbi.nlm.nih.gov/assembly/GCA_024336825
